# From Clinical Signs to Functional Recovery: A Practice-Oriented Review of Rotator Cuff Tear Management in Primary Care

**DOI:** 10.3390/jcm15176697

**Published:** 2026-08-29

**Authors:** Jakub Piotr Adamus, Kacper Dywan, Zuzanna Paszt, Julia Dąbrowska, Michal J. Tracz, Paweł Łęgosz, Sławomir Struzik

**Affiliations:** 1 Department of Clinical Immunology, Medical University of Warsaw, 59 Nowogrodzka Street, 02-005 Warsaw, Poland; jadamus.md@gmail.com; 2 Clinical Immunology Student Scientific Association, Medical University of Warsaw, 59 Nowogrodzka Street, 02-006 Warsaw, Poland; 3 Prof. M. Weiss Hospital, Mazovian Rehabilitation Centre “STOCER”, 12 Wierzejewskiego Street, 05-510 Konstancin-Jeziorna, Poland; 4 Department of Orthopedics and Traumatology, Medical University of Warsaw, 4 Lindleya Street, 02-005 Warsaw, Poland; zuziapaszt2006@gmail.com (Z.P.); pawel.legosz@wum.edu.pl (P.Ł.); 5 The Faculty of Medicine and Dentistry, Medical University of Warsaw, 61 Żwirki i Wigury Street, 02-091 Warsaw, Poland; j.dabrowska0312@gmail.com; 6 New York Medical College, 40 Sunshine Cottage Rd, Valhalla, NY 10595, USA; mtracz@montefiorenyack.org

**Keywords:** rotator cuff tear, general practice, primary care, physical examination, imaging, rehabilitation protocol, diabetes mellitus

## Abstract

Rotator cuff tear (RCT) is a leading cause of shoulder pain encountered by general practitioners. Population-based screening shows that nearly half of full-thickness tears are asymptomatic, allowing many lesions to progress undetected. This narrative review summarises 105 clinical, imaging, biomechanical and rehabilitation studies published in recent years. The review follows the disorder from early tendinopathy to full-thickness tear and discusses crucial risk factors (age, diabetes, hypercholesterolaemia, overhead work). It aims to guide early primary-care management of RCT. We examine the diagnostic accuracy of classic bedside tests, including the supraspinatus weakness and external rotation lag cluster. Furthermore, we explain when ultrasound or MRI adds value and what the classic findings are. An informative overview of patient-reported outcome tools (e.g., SPADI, WOSI, Constant–Murley, ASES) could help physicians select one for follow-up. The article objectively evaluates the evidence for conservative measures—structured exercise protocols, manual therapy, oral analgesics, and corticosteroid or platelet-rich plasma injections. Moreover, we propose an evidence-based postoperative rehabilitation timeline with milestones that incorporate targeted immobilisation and progressive loading. The article presents selected clinical situations, such as the impact of type 2 diabetes on tendon biology. We explore the viability of experimental adjuncts such as extracorporeal shock-wave therapy, low-intensity pulsed ultrasound and electrical stimulation. Finally, we compare recommendations from the American Academy of Orthopaedic Surgeons and the European Society for Surgery of the Shoulder and Elbow. We highlight similarities between the recommendations (e.g., overlapping clinical examination tests), thereby facilitating their clinical application and providing a pragmatic guide for general practitioners. Our synthesis draws on orthopaedic, radiographic and rehabilitation data to reduce diagnostic delay, optimise non-specialist resource use, and improve pain and functional outcomes for the growing population affected by rotator cuff tears.

## 1. Introduction

Shoulder pain is among the most common problems affecting patients in primary health care. It is estimated to affect around 70% of people during their lifetime, though estimates may vary across sources [1,2]. The incidence of shoulder pain is estimated at 14.7 per 1000 patients per year [3,4]. Despite this high burden, rotator cuff tears (RCTs) may remain under-recognised in primary care because symptoms are often non-specific and many full-thickness tears are asymptomatic [5,6]. Consequently, this review aims to provide a practical, primary-care-oriented framework for recognising suspected RCTs, selecting appropriate imaging and conservative treatment, and identifying patients who require timely specialist referral.

**Figure 1:**
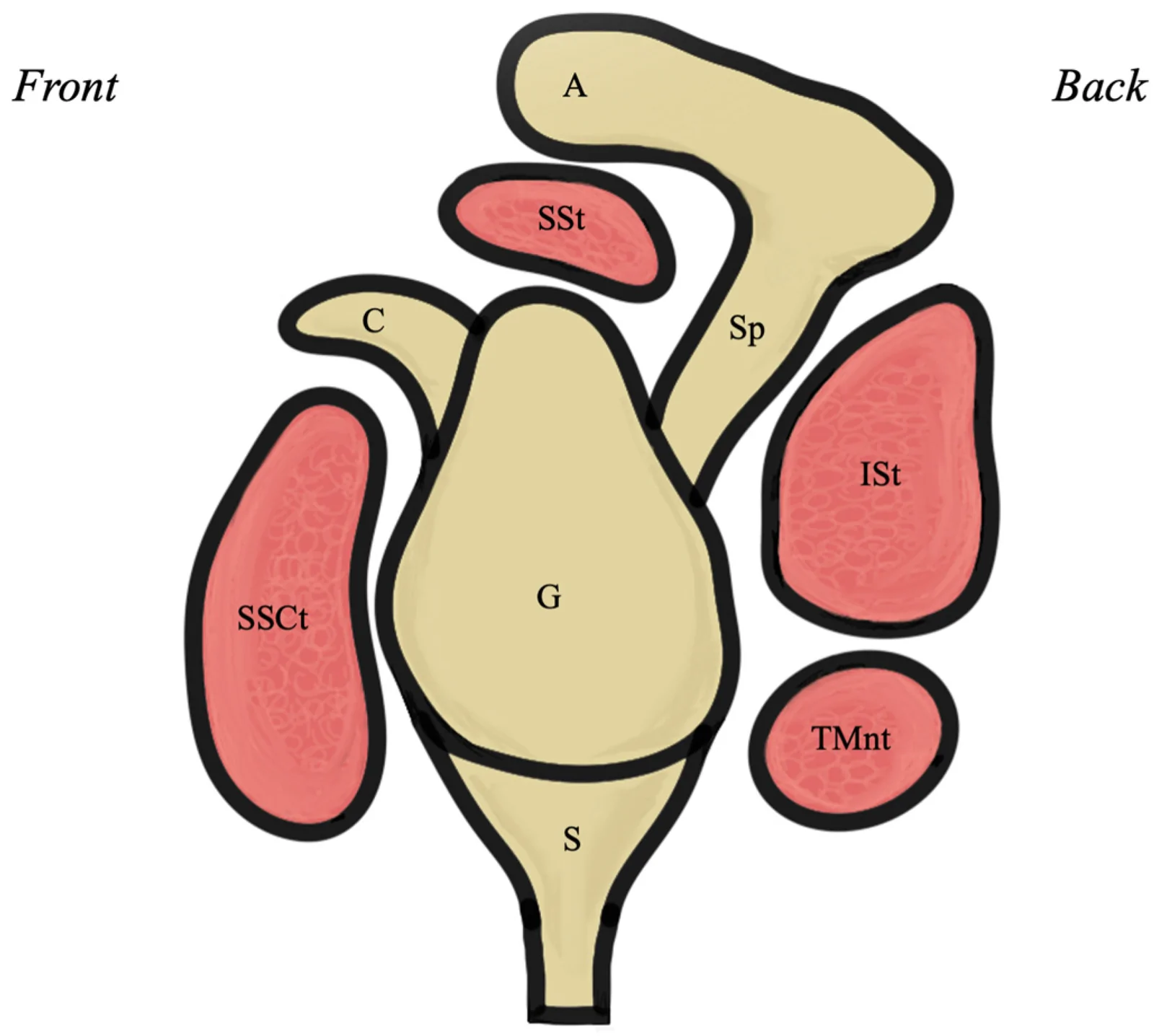
**Schematic of the left rotator cuff muscles in relation to the left scapula**. Lateral presentation. Left humerus is not presented. Marked structures: A—acromion, SSt—supraspinatus tendon, LHBt—long head of the biceps tendon, SSCt—subscapularis tendon, ISt—infraspinatus tendon, TMnt—teres minor tendon, H—humerus, HH—humeral head, L—labrum (glenoid labrum), G—glenoid, C—coracoid process, Sp—spine of scapula, S—scapula.

To achieve this, we first need to analyse shoulder anatomy to better understand the mechanisms underlying rotator cuff-related pain. The shoulder comprises three bones, the humerus, clavicle, and scapula, together with soft-tissue structures that connect them [7,8]. The glenohumeral joint is formed by the humeral head and the glenoid cavity. The articular surface is covered with cartilage, allowing smooth gliding movement [7,9]. The ligaments and joint capsule are passive stabilisers of the shoulder joint, while the rotator cuff (RC) muscles are the most important active stabilisers. This review pays particular attention to this muscle group. The RC consists of four muscles: supraspinatus, infraspinatus, teres minor and subscapularis [7,8,10]. Figure 1 presents various projections of the RC muscles, including their relationship to the scapula. The supraspinatus originates from the supraspinous fossa and inserts into the superior facet of the humeral greater tuberosity. The infraspinatus and teres minor arise from the infraspinous fossa and attach to the middle and inferior facets of the humeral greater tuberosity, respectively. The subscapularis originates from the subscapular fossa and inserts into the lesser tuberosity of the humerus. RC tendons blend, creating a unified structure near their attachment points [7,11,12]. These muscles are responsible for both external and internal rotation of the shoulder [13,14,15,16].

The mechanical properties of tendons are attributed to their collagen content, which makes them both flexible and strong enough to resist deformation [7,17]. Previous studies suggest that chronic tendinopathy causes altered collagen organisation and an inflammatory infiltrate. As a result, partial- or full-thickness tears can occur [5].

Studies have indicated that RCT injuries are associated not only with mechanical overload but also with chronic inflammatory and biochemical processes that contribute to tendon degeneration and pain. Previous studies have demonstrated the presence of pro-inflammatory cytokines and growth factors within the subacromial bursa of patients with RCTs [18]. Moreover, increased serum levels of tumour necrosis factor-α (TNF-α) were observed in patients undergoing arthroscopic surgery for RCT [19]. A clinical observational study demonstrated that elevated interleukin 1β (IL-1β) concentrations in the shoulder synovial fluid were associated with greater pain intensity and poorer functional outcomes in patients with chronic RCT [20]. These findings have been incorporated into clinical practice, where non-steroidal anti-inflammatory drugs (NSAIDs) are commonly used for the conservative management of RCT [21].

### Epidemiology and Patient Presentation

The exact prevalence of RC disease remains uncertain, as most individuals with RCT experience no pain or significant weakness and do not seek medical attention [5]. Large population studies show that almost one in two patients with a full-thickness RCT is completely asymptomatic (≈48–50%), meaning that a substantial proportion of tears remain undiagnosed until pain, weakness or loss of function prompts consultation [6]. The most significant risk factor for RCT is age [5,22]. A study found that the mean age of the 376 patients diagnosed with an RCT was 62.8 years [23]. A characteristic feature of RCT is its frequent bilateral occurrence. An earlier study showed that a full-thickness tear in the affected shoulder was associated with a 21% likelihood of a partial-thickness tear and a 36% likelihood of a full-thickness tear in the opposite shoulder [5,23]. Obesity, diabetes mellitus, and hypercholesterolaemia are regarded as pro-inflammatory conditions, which make them risk factors for RCT [5]. Chronic inflammation associated with RCT may result from both degenerative and traumatic mechanisms. Degenerative factors include ageing, bursal hypertrophy and repetitive microtrauma, while traumatic tears are commonly associated with acute shoulder injuries [24].

**Figure 2:**
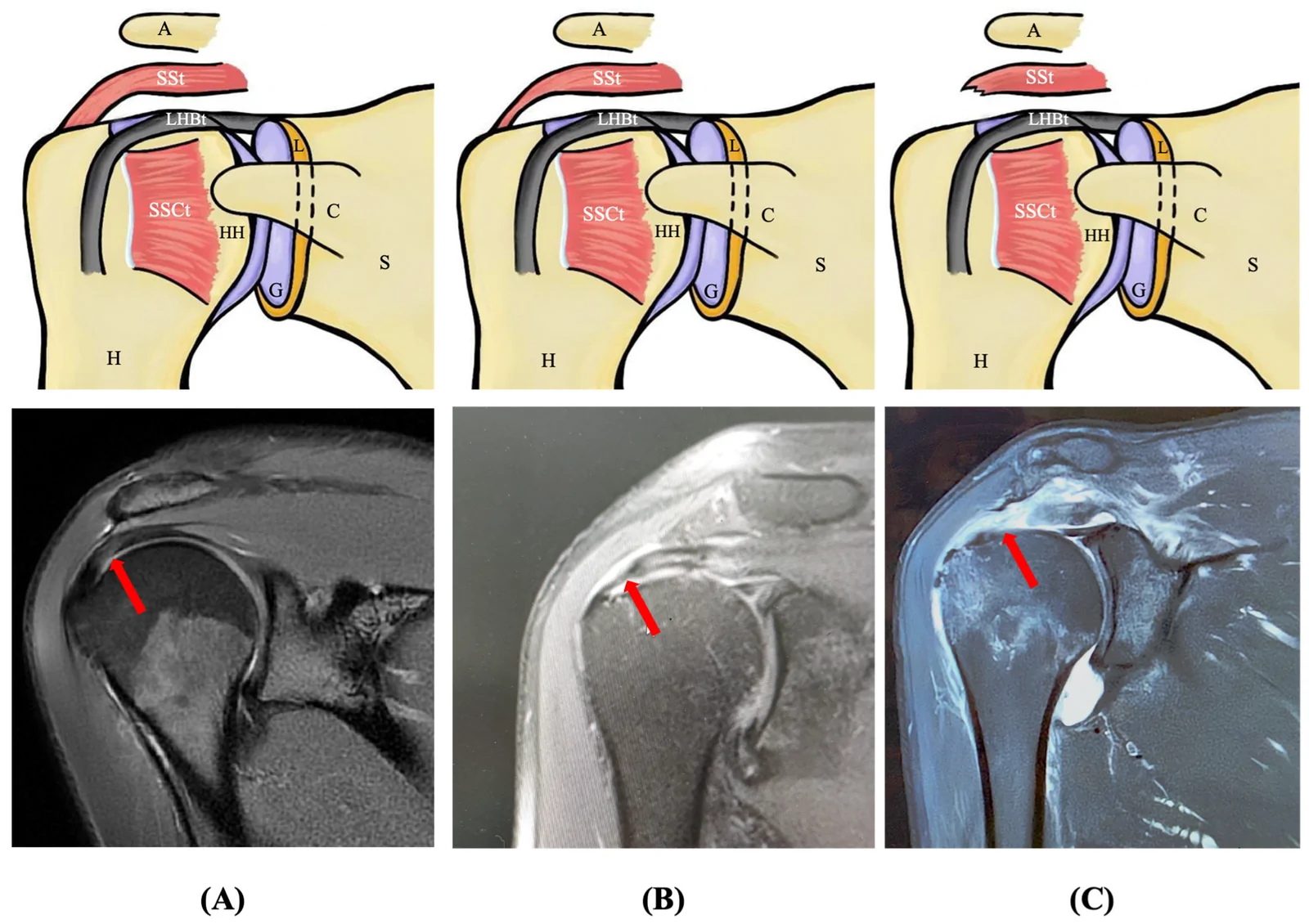
**Progressive pathology of the rotator cuff: MRIs ranging from intact anatomy to complete tear in the AP presentation**. Schematic and MRI-based visual comparison of the rotator cuff across three stages: (**A**) **intact rotator cuff**: normal tendon anatomy with preserved attachment and uniform tendon structure (red arrow); (**B**) **partial-thickness tear**: focal disruption of tendon fibres with preserved continuity and mild signal changes on MRI (red arrow points towards tendon thinning); (**C**) **full-thickness tear**: complete discontinuity of the supraspinatus tendon with tendon retraction, a fluid-filled gap, and superior humeral head migration evident on MRI (red arrow points towards the location where the tendon should be present). Each schematic is paired with a representative T2-weighted MRI image (oblique coronal plane), providing both anatomical and radiological perspectives. **Marked structures**: A—acromion, SSt—supraspinatus tendon, LHBt—long head of the biceps tendon, SSCt—subscapularis tendon, ISt—infraspinatus tendon, TMnt—teres minor tendon, H—humerus, HH—humeral head, L—labrum (glenoid labrum), G—glenoid, C—coracoid process, Sp—spine of scapula, S—scapula.

Apart from the tear size, the timing of diagnosis also plays a crucial role in determining prognosis [25]. Medium-sized tears show limited potential for spontaneous healing and may progress after injury [1,25,26]. Literature indicates that approximately 1% of adults present to primary healthcare each year with a first episode of shoulder pain [27,28,29]. A prospective longitudinal study found a significant risk of RCT progression over a short period. More severe tear types were most strongly associated with further enlargement [30]. The onset of pain may be a clinical indicator of tear progression [30]. This pathological progression is illustrated in Figure 2A–C, which presents a schematic and MRI-based visualisation of RC anatomy in its intact state, after a partial-thickness tear, and culminating in a full-thickness rupture.

This narrative review aims to compile clinically relevant evidence on how to identify, diagnose, conservatively manage, medicate, rehabilitate, and make referral decisions for patients with suspected or confirmed rotator cuff tears in primary care. Its main goal is to provide general practitioners with a practical guide to early detection, appropriate initial treatment, and prompt referral.

## 2. Materials and Methods

### 2.1. Literature Search Strategy

This narrative, practice-oriented review was designed to synthesise clinically relevant evidence on the diagnosis and management of RCTs in primary care. The literature search was completed in July 2025. Two independent researchers searched PubMed and the Cochrane Library for English-language articles published from 2010 onwards. The search used combinations of terms related to rotator cuff tears, rotator cuff tendinopathy, primary care, general practice, shoulder pain, physical examination, imaging, pharmacological treatment, physiotherapy, and rehabilitation. Examples of search phrases included “Rotator Cuff Tear Primary Care”, “Rotator Cuff Tear General Practice”, “Shoulder General Practice”, “Rotator Cuff Physiotherapy”, and “Rotator Cuff Tear Rehabilitation”. Following the database search, the reference lists of selected articles from PubMed and the Cochrane Library were manually screened to identify additional clinically relevant publications. This manual reference search was conducted for all selected full-text articles. Articles published before 2010 were included selectively if they provided essential background, historical context, or foundational information relevant to RCT diagnosis, epidemiology, clinical assessment, or management.

### 2.2. Inclusion and Exclusion Criteria

Articles were considered eligible if they addressed clinically relevant aspects of RCT or RC tendinopathy, particularly in relation to general practice or non-specialist clinical decision-making. Eligible publications included original clinical studies, systematic reviews, narrative reviews, and clinical guidelines. Studies were included if they discussed patient history, risk factors, physical examination, imaging, conservative treatment, pharmacological options, rehabilitation, or referral-related considerations. As this review was intended as a practical guide for general practitioners rather than a surgical review, surgical studies were included only when they provided information relevant to primary-care decision-making, postoperative rehabilitation, prognosis, or patient counselling. Studies focused exclusively on operative technique without clear relevance to primary care were not prioritised. Articles were excluded if they were not published in English, focused on paediatric populations, were conference abstracts or letters, or addressed unrelated shoulder disorders without a clear connection to RCT. Animal studies were generally excluded, except for selected evidence discussed in the section on external stimulation (please see Section 7.2), where preclinical data were used to contextualise emerging adjunctive therapies.

### 2.3. Study Selection

**Figure 3:**
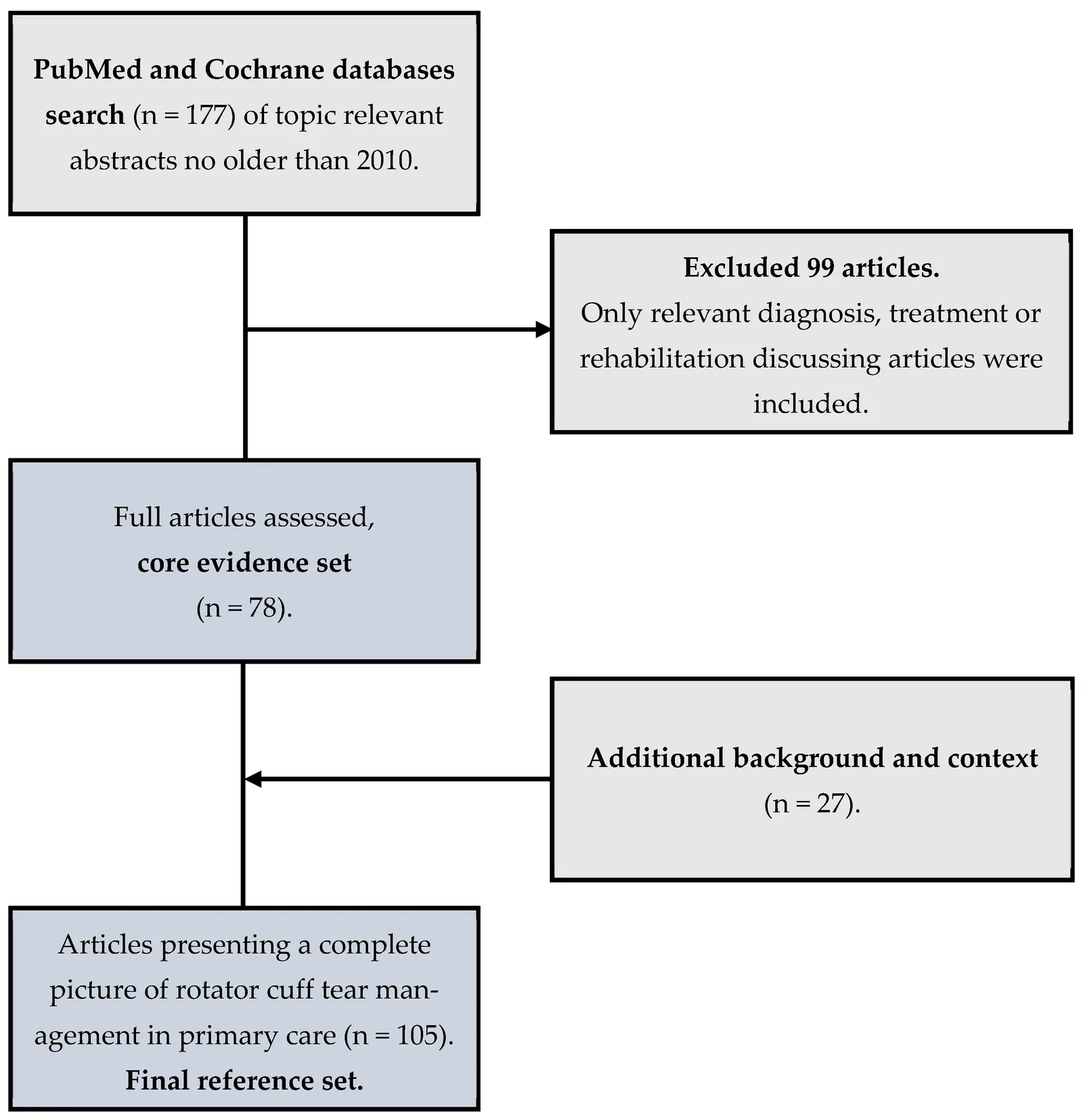
PRISMA-style flow chart presenting the selection of literature.

The database search identified 156 articles. Manual screening of reference lists yielded an additional 21 potentially relevant articles, bringing the total to 177 records for screening. No duplicates were identified. Two independent researchers screened the abstracts of all 177 records for relevance to the review’s aims. Disagreements were resolved through discussion with a senior author. After abstract screening, 99 articles were excluded for insufficiently addressing the clinically relevant diagnosis, treatment, rehabilitation, or primary-care management of RCTs. The remaining 78 articles were assessed in full and formed the core evidence set of this review. In addition, 27 older publications were included for background or historical value, bringing the total to 105 articles in the referenced literature. The study selection process is summarised in a PRISMA-style flow chart (Figure 3).

### 2.4. Data Charting

Data were charted narratively and thematically, consistent with the review design. For each included publication, the extracted information included the author, year of publication, relevance to general practice, and clinical relevance to RCT or rotator cuff tendinopathy. Depending on the focus of the source article, additional information was charted on physical examination, imaging, pharmacological treatment, conservative management, rehabilitation, surgical referral, postoperative care, guideline recommendations, or emerging therapies. The charted information was then organised into clinically oriented domains: epidemiology and risk factors, patient presentation, physical examination, imaging, pharmacological and conservative treatment, rehabilitation, postoperative considerations, special clinical scenarios, and guideline-based recommendations. Data were charted by one researcher and checked for accuracy and relevance by a second researcher.

## 3. Results

The diagnosis of an RCT is central to subsequent management. It involves taking a careful history, performing a physical examination, and conducting appropriate imaging tests. It is crucial to ask about previous injuries, sports, overhead activities, occupation, and problems with activities of daily living. The two main causes of RCT are degeneration (more common) and injury (e.g., acute tears) [31]. Risk factors to be identified include advanced age, diabetes, smoking, body mass index, and osteoporosis [32]. Moreover, RCTs are more often seen in females and in the dominant arm [31,33]. A significant proportion of patients with cuff tears remain asymptomatic, a finding that occurs twice as often as in those with symptoms [32]. Patients with RCT report shoulder pain, especially at night. The onset of pain is often unknown, but some may report a pop in the shoulder or back, which may be associated with an acute tear [34].

### 3.1. Physical Examination

**Figure 4:**
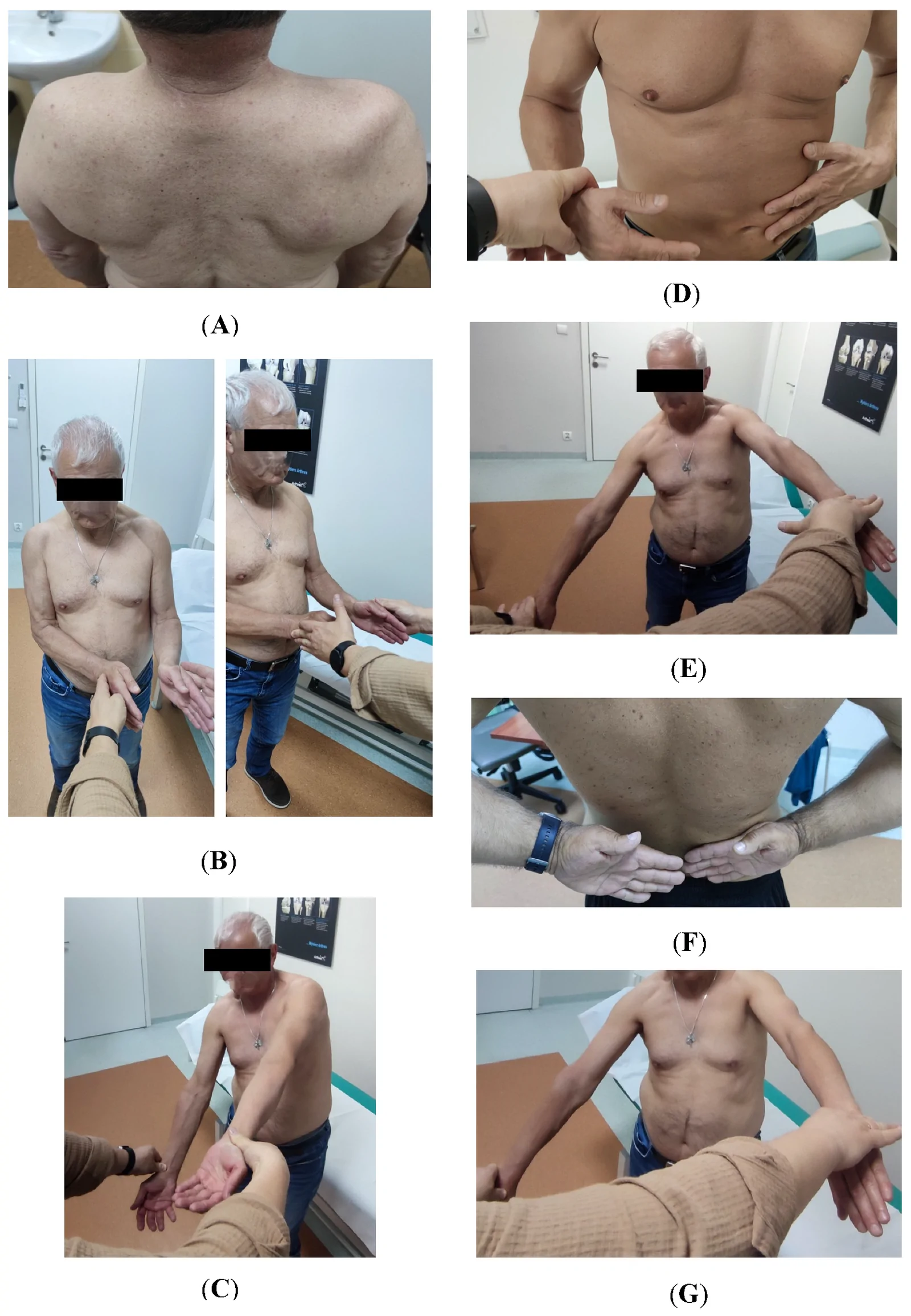
**Physical presentation of a damaged rotator cuff with selected physical examination tests.** (**A**) **Muscle atrophy** in the right supraspinatus and infraspinatus fossae due to a massive rotator cuff tear. No atrophy is observed on the left side. (**B**) **External rotation resistance test** from two perspectives, right arm positive, left arm negative (normal). (**C**) **Speed test**, right arm positive, left arm negative (normal). (**D**) **Belly press test**, right arm positive, left arm negative (normal). (**E**) **Drop arm test**, right arm positive, left arm negative (normal). (**F**) **Lift-off test**, right arm positive, left arm negative (normal). (**G**) **Empty can test**, right arm positive, left arm negative (normal). Video presentations of the belly press test, bear hug test, lift-off test, and empty can test are available in the Supplementary Materials.

A patient with suspected RCT should be appropriately undressed to allow the doctor to conduct the best possible assessment of the shoulder girdle, upper limbs, and spine. Muscle atrophy is assessed by palpation, noting any reduction in muscle mass and comparing it with the opposite side [35]. Figure 4A shows atrophy of the right supraspinatus and infraspinatus muscles. RC weakness is uncommon; as a result, strength tests of the RC and scapular muscles may be normal. The examiner also rules out radicular pain originating from the spine [34]. In cases of massive RCTs, superior displacement of the humeral head may be observed, with the humeral head abutting the acromion. Subsequently, active and passive range of motion (ROM) and tenderness are evaluated. ROM is measured in degrees and is best assessed with a goniometer [35]. Pain may occur within the ROM of 90° to 120° during flexion or abduction [34]. Nevertheless, symptoms such as pain or loss of range are not specific to RCT [35]. A traumatic full-thickness cuff tear presents with oedema and inflammation [32].

Several physical examination tests may be performed. The speed test (Figure 4C) is a useful clinical manoeuvre for assessing the long head of the biceps tendon and identifying possible biceps tendinopathy or superior labral lesions. The patient’s shoulder is flexed to 90°, the elbow fully extended, and the forearm supinated. Downward resistance is applied to the forearm while the patient maintains this position. A positive result is indicated by pain localised to the bicipital groove, suggesting pathology of the biceps tendon or labrum [36]. The drop-arm test (Figure 4E) has high specificity but low sensitivity [37]. With the patient’s arm fully extended, the examiner passively abducts it to 90° to achieve full external rotation while supporting the elbow. Thereafter, the examiner removes the elbow support and instructs the patient to gradually lower the arm back to a neutral position. The test is considered positive if there is an abrupt loss of arm control or noticeable weakness in maintaining arm position during the eccentric phase of abduction. Additionally, pain when lowering the arm may indicate a full-thickness supraspinatus tendon tear [35]. Lift-off, i.e., the Gerber test (Figure 4F), is used to screen for subscapularis tears. To conduct the test, the patient must be standing (or seated). Next, the examiner places the dorsum of the patient’s hand on the mid-lumbar spine, then asks the patient to actively “lift” the hand straight posteriorly (away from the back) while the examiner steadies the scapula. Inability to raise the hand, a lag when the physician’s support is withdrawn, or substitution with elbow/shoulder extension is a positive result. This may indicate subscapularis rupture or marked weakness. Some data suggest a very high specificity (>90%) but modest sensitivity (18–35%), so a positive test is best used to confirm, not to rule out, a clinically significant subscapularis pathology [38,39,40]. Jobe’s test, also known as the supraspinatus test or empty can test (Figure 4G), is used to assess lesions of the supraspinatus tendon. The patient’s arm is actively elevated in abduction to 90°. With the patient’s hand clenched into a fist and the thumb extended, the shoulder is actively internally rotated and positioned at a 30° forward angle within the scapular plane, ensuring the thumb is directed downward (empty can position). The examiner applies downward pressure on the abducted arm to assess resistance. This test is considered positive if there is evident weakness against resistance when the arm is abducted at 90°, compared with a 30° forward position, suggesting possible supraspinatus pathology [35]. To facilitate visualisation of the tests discussed in this chapter, we prepared a series of short videos. They show the tests being performed in both healthy and RCT-affected shoulders and are available in the Supplementary Materials.

Several significant scales have been developed to assess shoulder-related pain, disability, and functional limitations. These include the Shoulder Pain and Disability Index (SPADI), the Western Ontario Shoulder Instability Index (WOSI), the Constant–Murley Score (CMS), and the American Shoulder and Elbow Surgeons questionnaire (ASES) [41,42,43,44]. These instruments differ in scope, structure, and feasibility in everyday clinical practice; however, their reliability, validity, and responsiveness have been evaluated in previous studies, supporting their use in monitoring shoulder-related outcomes [45,46].

The SPADI was designed to assess pain and functional impairment associated with shoulder pathology. It is a self-report index comprising 13 items, organised into two subscales: pain (5 items) and disability (8 items) [44]. Previous studies have demonstrated that the SPADI is a reliable, valid, and responsive patient-reported outcome measure for patients with shoulder disorders, making it suitable for monitoring changes in symptom severity and function over time [44,45]. Each item is scored on a Visual Analogue Scale (VAS) from 0 (no pain or difficulty, the best condition) to 10 (the worst imaginable pain or extreme difficulty requiring assistance, the worst condition) [44]. The VAS component should be interpreted primarily as a pain-intensity measure rather than a comprehensive shoulder function score.

The Western Ontario Shoulder Instability Index is a self-report scale that reflects the current condition of patients with shoulder instability [43]. The WOSI comprises 21 items, grouped into four domains. The first domain, addressing physical symptoms, includes 10 items. The remaining domains are sports, recreation, and work (4 items), lifestyle (4 items), and emotional well-being (3 items). The total score ranges from 0 to 2100, with 0 indicating no impairment in shoulder-related quality of life and 2100 indicating severe impairment [47]. WOSI has demonstrated reliability, validity, and responsiveness in patients with shoulder instability; however, it is not specific to rotator cuff tears. Therefore, in the context of RCT, it should be interpreted as a broader shoulder-related quality-of-life measure rather than an RCT-specific assessment tool [43,45,47].

The available evidence does not support the Constant–Murley Score as the gold standard for shoulder assessment. Although it is recommended for subacromial pathology, a standardised evaluation of its properties across different shoulder conditions has yet to be established [42,45]. It is the most concise assessment tool, incorporating both self-reported and examiner-based evaluations and combining their data into a single total score [42,45]. The CMS scale evaluates four key aspects of shoulder pathology: two subjective (pain and activities of daily living) and two objective (ROM and strength). The subjective components can be awarded up to 35 points, while the objective components account for 65 points, leading to a maximum total score of 100, indicating optimal shoulder function. Pain and ADL are assessed through patient self-reporting, whereas ROM and strength require a physical examination conducted by an orthopaedic surgeon or physiotherapist [42]. Cross-cultural adaptations and validation studies further support the use of CMS in shoulder assessment, although examiner-dependent components, such as ROM and strength, require standardised administration to improve reproducibility [45,48].

The ASES is a comprehensive assessment tool designed for both patients and examiners, featuring a relatively large number of items, which may be time-consuming for clinicians [45]. However, research indicates that the ASES is a reliable, valid, and responsive measure of outcomes [46]. Therefore, the ASES may be particularly useful when a more detailed assessment of pain and shoulder function is needed, whereas shorter patient-reported tools may be more feasible in time-limited primary care consultations [45,46].

### 3.2. Imaging

Magnetic resonance imaging (MRI) and ultrasound (US) are the most essential imaging modalities for diagnosing RCT. Multiple studies have shown that US and MRI have comparable accuracy before surgery, although US precision depends more on the operator and available equipment [49].

Ultrasound shows soft tissue and provides real-time information about muscles and tendons. Furthermore, it allows for visualisation in various positions. When evaluating accuracy, cost, and safety, ultrasound is the most optimal choice [50].

Magnetic resonance imaging, the gold standard, reveals soft tissues and can visualise whether an RCT is present and whether it is partial or full thickness. MRI evaluates the extent of tendon involvement in RCT, as well as retraction, fatty infiltration, and muscle atrophy [32]. Fatty infiltration has been identified as a negative prognostic factor for cuff tendon repair. The Goutallier classification is commonly used to assess fatty infiltration of the RC musculature. The grading system comprises five stages, from 0 (normal muscle without fatty infiltration) to 4 (more fat than muscle) [51,52]. When comparing the degree of fatty degeneration, radiologists consistently noted that MRI and CT findings were comparable; however, they showed only moderate consistency in identifying the same level of muscular degeneration on MRI as observed on CT. A comparison of cross-sectional area with the grade of fatty degeneration showed a highly statistically significant correlation between the extent of atrophy and the degree of fatty degeneration [53]. MRI provides information about the tear pattern, which influences decisions about which surgical techniques should be used. In a full-thickness RCT, there are considerable differences in retraction distances between delaminated and non-delaminated tendons. It is crucial to recognise and document delaminated tendons, as they are more likely to fail conservative treatment and require surgical intervention [54]. Full-thickness tears are characterised on MRI by a discontinuity of the tendon on T1-weighted and PDFS images, which appears brighter on T2-weighted images [55]. Patients with high clinical suspicion of full-thickness RCT but negative US should undergo MRI for more detailed assessment of RC integrity. Ultrasound is less reliable for detecting partial-thickness RCT [49].

Magnetic resonance arthrography (MRA) is an MRI of a joint performed after injection of a contrast medium. Studies have shown no significant difference in diagnostic performance between Three-Dimensional Isotropic and Two-Dimensional Conventional Indirect MRA for diagnosing RCT [56]. A meta-analysis found that MR arthrography is the most sensitive and specific method for diagnosing both full- and partial-thickness RCT, whereas ultrasound and MRI show comparable sensitivity and specificity [57].

X-rays are primarily used to visualise calcifications and to rule out degenerative bone disease or bone damage. In RC calcific tendinopathy, calcific deposits appear as high-density opacities near the humeral head on X-ray (AP, outlet and axillary views), as hyperechoic foci on US, and as signal voids on MRI [58].

To describe rotator cuff tears, the acronym PEARL may be utilised. Pattern (P), extension (E), fatty atrophy (A), retraction (R), and location (L) are documented [59].

## 4. Clinical Findings

The aetiology of RCT can be both traumatic and degenerative, with degenerative changes being more widespread. Repetitive exposure to internal and external factors causes damage that ultimately leads to a full-thickness tendon tear [32]. The most common symptom, which can develop at any stage of RCT, is shoulder pain, often during overhead activities. The pain may occur at night, leading to sleep disturbances [5]. However, some patients do not experience pain and maintain normal shoulder function [5,60]. Commonly reported symptoms include weakness and reduced ROM, both of which negatively impact quality of life [61,62,63]. There is a well-defined pathophysiological progression from tendinopathy to partial-thickness tears and, eventually, full-thickness tears. However, the severity of symptoms does not always correspond to the extent of histopathological changes [5]. The cited article reports pseudoparalysis, characterised by the inability to actively flex the arm above 90° and to hold it at 90° [62,64] in patients with RCT.

## 5. Treatment

The primary goal of treatment is to reduce pain and improve upper-limb function [5]. Physical therapy is among the most commonly recommended interventions. A study of 381 patients with a mean age of 62 found significant improvements in patient-reported outcomes and a reduced need for surgery after physical therapy [5,65]. Even in cases of massive tears, a rehabilitation programme targeting the deltoid muscle effectively improves function and reduces pain [5,66]. Pharmacological treatment, including acetaminophen, NSAIDs, and corticosteroids, can reduce pain in patients with symptomatic RCT; however, their effects are short-term [5,21,67].

A frequently used treatment for shoulder pain is glucocorticoid injection, which provides rapid pain relief and acceptable anti-inflammatory effects. In a series of reviews, steroid injections demonstrated greater effectiveness than physiotherapy and systemic NSAIDs [68,69,70,71]. However, a study revealed that patients who received steroid injections developed RCT more frequently than those who did not, with a mean follow-up of 39 months [70]. The use of platelet-rich plasma (PRP) was reported to have a similar effect on pain relief and ROM improvement to steroid injection at all follow-up time points [72].

A recent network meta-analysis compared three commonly used injection-based conservative treatment modalities for RCT [73]. It was demonstrated that hyaluronic acid yields favourable outcomes in reducing pain without significant side effects. However, these benefits were observed only in the short term [73,74]. PRP was injected to promote tendon-to-bone healing by delivering a high concentration of platelets and growth factors [73,75]. The article reported better long-term pain relief, with results comparable to those achieved with physical therapy [73,76]. The third and most commonly used injection therapy in the cited meta-analysis was corticosteroid injection. Despite its beneficial short-term pain-relieving effects, its therapeutic efficacy and safety profile may be inferior to those of the two previously described injection therapies [73].

Table 1 comprehensively summarises pharmacological management alternatives in RCTs, useful for primary care physicians.

From a primary-care perspective, referral to an orthopaedic specialist should be considered when symptoms persist despite an adequate trial of conservative treatment, when pain continues to substantially affect sleep or quality of life, or when weakness and functional limitation impair activities of daily living or work. Earlier referral is particularly appropriate for traumatic tears, suspected large or progressive full-thickness tears, pseudoparalysis, marked loss of strength, or imaging features associated with a lower likelihood of successful non-operative management, such as tendon retraction, delamination, fatty infiltration, or muscle atrophy [25,54,64].

In a substantial number of patients, surgical intervention becomes necessary at some stage in the disease course. Surgery can be performed using direct open, arthroscopic-assisted mini-open repair, or all-arthroscopic techniques [32]. A previous study showed that early identification of medium-, large-, or massive-sized RCTs in primary healthcare may result in better structural integration during surgery and improved postoperative outcomes [25]. Moreover, surgical repair of RCTs often markedly alleviates shoulder pain. However, the procedure itself carries a significant risk of re-tear [77]. Nevertheless, biological augmentation has emerged as a promising approach to enhance the healing of repaired RCTs [77,78].

Both the European Society for Surgery of the Shoulder and Elbow (SECEC) and the American Academy of Orthopaedic Surgeons (AAOS) provide recommendations for the diagnosis and management of RCT. A comparison of the guidelines issued by these organisations is presented in Table 2.

**Table 1:** **Pharmacologic** **options in RCT management: primary care overview.**

Therapy	Mechanism of Action	Onset	Duration	Evidence of Tendon-Related Harm	Role in Primary Care	JBJS Levels of Evidence (I–V)	References
**NSAIDs**	COX inhibition, anti-inflammatory	Rapid	Short-term	*None reported*	**First-line analgesia** with caution	I	[67]
						II	[68]
**Acetaminophen**	Central COX inhibition (weak)	Rapid	Short-term	*None reported*	**Safe adjunct** or NSAID alternative	V	[5]
**Corticosteroid** **injection**	Potent anti-inflammatory, reduces oedema	24–72 h	2–6 weeks	**Yes**, risk of tendon degeneration with repeated use	**Reserved**; limited to 1–2 injections	II	[69]
						III	[70]
						II	[71]
						II	[72]
						II	[73]
**Platelet-rich** **plasma**	Growth factor release, healing modulation	~1 week	Up to 12 weeks	*None reported*	**Emerging therapy;** not routine in primary care	II	[72]
						II	[73]

**Table 2:** **Comprehensive summary of the core information from AAOS and SECEC recommendations. Navy blue** highlights similarities between the two organisations’ recommendations, including selected tests presented in Figure 4B–G and in the Supplementary Materials.

Feature	American Academy of Orthopaedic Surgeons (AAOS)	European Society for Surgery of the Shoulder and Elbow (SECEC)
**Clinical examination**	**A combination of tests increases diagnostic accuracy:** **(1) Bear hug test**, **(2) belly press test**, **(3)** empty can test, **(4)** external rotation lag sign, **(5)** external rotation resistance test, **(6)** full can test, **(7)** Hawking test, **(8) Jobe test**, **(9)** hug up test, **(10)** internal rotation resistance test, **(11)** lateral Jobe test, **(12) lift off test**, **(13)** NEER test, **(14)** Patte test, **(15)** Yokum test.	**Aspects that should be performed:** **(1)** Inspection, **(2)** active and passive range of motion, **(3)** strength of the different muscles of the RC, **(4) Jobe test**, **(5) lift off test**, **(6) belly press test**, **(7) bear hug test**, **(8)** Hornblower’s test.
**Imaging**	– Ultrasound, a useful tool in diagnosis of RCT, but negative result does not exclude RCT. – MRA and MRI is helpful in diagnosis of RCT, but effectiveness is variable.	– Ultrasound should be performed as a first-line investigation. – MRI or CT should be performed when planning a surgery.
**Prognostic factors**	**(1) Age**, **(2)** diabetes mellitus, **(3) worker’s compensation**, **(4) comorbidities**, **(5)** higher BMI, **(6)** patients’ expectations.	**(1) Age**, **(2)** gender, **(3) comorbidities**, **(4)** smoking, **(5)** sports, **(6)** pain, **(7)** prior treatment, **(8)** sleep disturbances, **(9)** functional limitations, **(10)** occupations, **(11) workman’s compensation**, **(12)** hand dominance, **(13)** traumatic aetiology.
**Treatment success**	*No information*	**Should be defined as:** (1) Improved pain management (2) Improved range of motion (3) Improved strength (4) Improved quality of life (5) Patients satisfaction with treatment
**Follow-up**	*No information*	6 to 12 months after treatment
**Management**	– Compares the effectiveness of various treatments, including both surgical and conservative management. – Provides treatment recommendations based on the severity of the injury.	*No information*

The SECEC and the AAOS jointly consider clinical examination essential and include the tests listed in the table. Both organisations also highlight the invaluable role of imaging in diagnosing RCT. The AAOS describes the effectiveness of US, MRA, and MRI in diagnosing RCT. The SECEC considers US the first-line diagnostic tool, while MRI and CT are deemed valuable in preoperative planning. The SECEC, together with the AAOS, identifies risk factors for RCT that should be considered during the patient history, as they can influence treatment effectiveness. The SECEC outlines how treatment success should be defined and for how long patients should be followed up with, whereas AAOS guidelines do not contain those specifics. The AAOS, in contrast to the SECEC, emphasises a broader approach to the treatment of RCT. It focuses not only on the severity of the tear and patient-specific factors but also on the evidence supporting various treatment modalities [79,80].

## 6. Rehabilitation

Rehabilitation is an essential component of managing RC pathologies, regardless of whether a conservative or surgical approach is selected. By itself, it can resolve a significant proportion of habitual pathologies. For surgical procedures, rehabilitation plays a crucial role not only in the postoperative period but also, in many cases, preoperatively [81], thereby optimising outcomes for RC patients undergoing surgical intervention. Its primary objectives include reducing pain and improving ROM, strength, function, and mobility [82,83]. Indications for conservative treatment include RCT without rupture, degenerative tears, partial tears involving less than 50% of the tendon thickness, and chronic full-thickness tears in elderly patients [81,84]. Therapeutic exercise should be prioritised for patients with degenerative RCT, as surgical intervention has not demonstrated clinically superior outcomes compared with non-surgical treatment in this population [84]. Current evidence strongly supports exercise therapy as the first-line treatment for patients with RCT [83].

### 6.1. Post-Operative Rehabilitation

The aims of postoperative RCT rehabilitation are to restore full, symmetrical passive and active ROM, to provide balanced strength across the glenohumeral and scapulothoracic joints, and to achieve pain-free shoulder function [85]. However, most studies rarely detail the surgical procedure [86], a crucial factor in postoperative rehabilitation. Physiotherapists should focus on restoring joint mobility, shoulder function and muscle strength. The current literature lacks robust evidence supporting a clear correlation between postoperative rehabilitation protocols and RCT size [81]. There is a discrepancy between adherence to the biological healing time of the repaired tendon [87] and the use of a milestone-based protocol [86]. A UK survey found that individual patient factors are more important than time-based benchmarks [86]. Although the AAOS and the American Society of Shoulder and Elbow Therapists (ASSET) propose a five-phase protocol depending on the size of the tear and the quality of the tendon tissue [85], the timeframe is intended to allow for healing and procedures by avoiding excessive pressure and tension on the repaired structures [86]. However, the rehabilitation protocol should be tailored to each patient [81].

### 6.2. Manual Therapy

Manual therapy (MT) is reported to relieve pain through a hypoalgesic effect or to improve shoulder biomechanics by increasing ROM in the affected area [82]. It is often used alongside exercise to help restore ROM and function in patients with partial RCT or degenerative changes [82]. Physiotherapists can apply MT to the shoulder girdle, cervical and thoracic spine to help modulate nociceptive stimulation and increase exercise tolerance [83].

Evidence for the use of MT alone is lacking, and evidence from RCTs regarding the addition of MT to exercise treatment is inconclusive [88]. In a small RCT, MT may delay or eliminate the need for surgery when combined with a strengthening exercise programme [82]. Studies debate whether there is a significant advantage to adding MT to exercise for pain reduction at rest and during movement, or for improved function in short- and long-term outcomes [88].

### 6.3. Exercises

Exercise-based rehabilitation should be a priority in the management of RC repair to optimise recovery. Early implementation of ROM exercises has been shown to accelerate tissue healing, reduce postoperative stiffness, and not increase the risk of re-tear [87]. Despite this, considerable variability exists in rehabilitation protocols, and no standardised approach is universally recognised as superior [89]. A critical challenge in postoperative rehabilitation is balancing early mobilisation to prevent joint stiffness with the need to minimise mechanical stress on the repaired tendon. While early passive or active ROM exercises can increase shoulder mobility, they may also increase the risk of re-tear compared with delayed motion [81]. Current evidence supports a progressive approach, beginning with passive ROM exercises in the first 6 weeks postoperatively and transitioning to active movements once the repair has achieved sufficient structural integrity. Resistance training usually lasts 12–16 weeks, and return-to-sports and work-specific rehabilitation begins after 4–5 months, depending on individual patient factors [87]. The optimal exercise strategy remains a matter of debate, as the existing literature offers no consensus on whether rehabilitation should focus primarily on RC strengthening, using conventional resistance exercises such as external and internal rotation and arm elevation, or emphasise alternative muscle groups to minimise RC loading, including closed kinetic chain elevation, shoulder muscle re-education, assisted arm raises and scapular control. Most rehabilitation protocols integrate both approaches, although there is insufficient evidence to determine which is superior [84]. Some rehabilitation protocols recommend gently assisted exercise as early as the second postoperative week, in contrast to conservative approaches [87].

Given the complexity and individualised nature of rehabilitation, a multidisciplinary approach involving primary care physicians, orthopaedic surgeons and physiotherapists is essential for developing personalised rehabilitation pathways that improve clinical outcomes and functional recovery [86]. Table 3 presents the proposed optimised postoperative rehabilitation timeline for patients who underwent RC repair.

### 6.4. Immobilisation

Post-operative immobilisation is a standard component of care following RC repair, with its duration often tailored to tear size and surgical technique. Most surgeons recommend using a sling or abduction pillow for 4–6 weeks [86]. ASSET recommends strict immobilisation for 2 weeks, followed by passive ROM at 2 weeks and active-assisted ROM at 6 weeks for small and medium tears, while recommending 6 weeks of strict immobilisation for large and massive tears [85]. Some studies have shown that immobilisation can support tendon healing, improve limb function, and enhance collagen organisation within four weeks, with improved mechanical properties observed after sixteen weeks [90,91,92].

However, prolonged immobilisation is also associated with complications such as reduced ROM, shoulder stiffness, and an increased risk of adhesions [81,87,93], as well as alterations in tissue composition, including changes in water and glycosaminoglycan concentrations, collagen cross-linking, fat infiltration, and fibre orientation [83]. Notably, a six-month follow-up found that patients who underwent strict immobilisation had reduced flexion, abduction, and external and internal rotation compared with those who began early passive or active motion [81]. Physiotherapists often favour more aggressive rehabilitation protocols with shorter immobilisation and earlier strengthening [94], particularly in younger patients with small tears. In contrast, delayed rehabilitation may be more appropriate for older individuals at greater risk of re-tearing [93].

Regardless of the chosen protocol, patient education remains essential. Clear guidance on the duration of immobilisation, permitted activities, and adherence to home exercises is crucial for achieving optimal outcomes and minimising the risk of repair failure [86].

## 7. Additional Clinical Considerations

### 7.1. Diabetes Mellitus Type 2

Diabetes mellitus (DM) type 2 is a chronic disease that affects multiple organs, including the musculoskeletal system. Although the exact cause remains uncertain, patients typically exhibit increased inflammation, elevated glucose levels, and stiffer connective tissue, which can ultimately limit shoulder motion [95,96]. The chronic inflammatory process in DM has been linked to excessive deposition of advanced glycation end products, resulting from prolonged hyperglycaemia [97,98]. These changes contribute to the higher prevalence of RCT and even re-tearing in the DM population [96,97,99].

Furthermore, DM patients are more prone to tendinopathy and to poor-quality bone and tendon healing [96,99]. These alterations likely stem from insufficient collagen formation, increased calcification, and tendon thickening [99]. Researchers have extensively explored the link between DM and RCT, but most data still come from animal studies. Given these clinical implications, clarifying the underlying biology is essential—we need it to develop therapies that relieve shoulder complications in people with DM and, ideally, prevent them altogether [97].

### 7.2. External Stimulation

Advances in medical technology have prompted investigators to seek new strategies to enhance bone and tendon repair after RC surgery. Physical forms of external stimulation are candidates, yet clinical evidence remains sparse. Several studies have investigated modalities such as extracorporeal shock waves (ESWs) and low-intensity pulsed ultrasound (LIPUS) for their potential therapeutic benefits. Experimental work suggests that low-energy ESWs can stimulate new vessel formation, reduce oedema and increase cellular ingrowth within the tendon, changes that could speed recovery [100,101].

To date, however, the supporting data are limited to animal models, and their relevance to humans remains to be confirmed. Similarly, LIPUS appears to promote angiogenesis, facilitate tendon repair and temper inflammation [101,102,103]. As with ESWs, evidence for LIPUS rests largely on animal studies; rigorous human trials have yet to be conducted.

Low-frequency electrical stimulation has also been investigated, with reports of increased collagen synthesis, cellular proliferation, and vessel formation [101,104]. While these approaches are promising adjuncts after surgery, well-designed clinical trials are needed to confirm both their safety and their effectiveness.

## 8. Conclusions

Shoulder pain is one of the most common complaints in primary care, and rotator cuff tears (RCTs) are a frequent underlying cause that may still be overlooked. These tears result from traumatic incidents or, more commonly, from degenerative changes due to repetitive stress. While the most characteristic complaint is pain, especially during overhead activity or at night, patients may also experience weakness, reduced range of motion, and decreased quality of life. Notably, the level of pain rarely correlates with the actual size of the tear. By integrating multidisciplinary evidence, this review provides practical guidance to shorten diagnostic delays, streamline resource use in non-specialist settings, and enhance pain relief and functional recovery for the growing population affected by RCT.

Accurate treatment starts with an exact diagnosis, which relies on the history, the physical exam and appropriate imaging. Validated tools, such as the SPADI, help assess how the injury limits daily activities. Whether the plan is conservative or surgical, rehabilitation remains at the centre of care, aiming to restore movement, strength and full shoulder use. Conservative management is usually recommended for partial tears and for older patients with long-standing full-thickness injuries. Structured exercise remains the main part of non-operative care; manual techniques provide, at best, a modest incremental benefit.

After surgical management, introducing gentle passive and isometric movements early in rehabilitation appears to aid recovery without increasing re-tear rates. Even so, rehabilitation programmes vary widely, and no protocol is regarded as universally superior. Therefore, care plans should be tailored to each patient and formulated in collaboration with the general practitioner, orthopaedic surgeon and physiotherapist. Immobilisation strategies likewise differ and should be adjusted to the patient’s age, tear size, and the repair method used. Teaching patients about their condition remains vital for good adherence and outcomes.

While research is advancing biological approaches to enhance tendon-to-bone healing, we still need more clinical trials to confirm their effectiveness and long-term safety.

**Limitations:** Although the literature selection process was conducted by two independent researchers and transparently summarised using a PRISMA-style flow chart, study inclusion was guided primarily by clinical relevance. Because this is a narrative review rather than a systematic review or meta-analysis, formal risk-of-bias assessment, certainty-of-evidence grading, and quantitative pooling of outcomes were not performed. In addition, the reviewed literature spans primary care, orthopaedics, radiology, pharmacology, and rehabilitation, introducing heterogeneity in study populations, diagnostic standards, treatment protocols, and healthcare settings. Therefore, the proposed recommendations should be interpreted as a practical clinical framework that may require adaptation to local imaging access, physiotherapy availability, and referral pathways.

**Strengths:** A major strength of this review is its direct applicability to primary care, where shoulder pain is common, and RCTs may be under-recognised initially. By integrating evidence on history-taking, physical examination, imaging, pharmacological options, conservative treatment, rehabilitation, and referral-relevant considerations, the article provides a comprehensive yet clinically accessible guide for non-orthopaedic physicians. Additional strengths include high-quality original figures, summary tables, MRI-based illustrations, suggested postoperative rehabilitation milestones, and Supplementary Videos demonstrating selected shoulder examination tests, all of which enhance the manuscript’s educational and practical value. The comparison of AAOS and SECEC recommendations further strengthens the review by translating specialist guidance into a more usable framework for everyday general practice.

## Figures and Tables

**Table 3:** **Suggested** **postoperative rehabilitation milestones after rotator cuff repair.**

	Timeframe	Focus	Activities	References
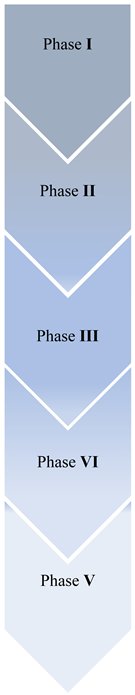	0–2 weeks	Protection, healing initiation	Sling/abduction pillow, education, pendulums (if allowed)	[85,86]
	2–6 weeks	Passive ROM, avoid stress	Passive elevation, external rotation < 30°, isometrics (if tolerated)	[86,87]
	6–12 weeks	Active-assisted ROM, control muscle imbalance	Wall walks, cane-assisted flexion, scapular setting	[81,85,86]
	12–16 weeks	Strengthening (light resistance)	Resistance bands: - external rotation, - deltoid re-education	[81,85]
	16+ weeks	Functional/sport-specific training	Overhead movements, plyometrics, return to work/sports	[81,85,87]

## Data Availability

No new data were created or analyzed in this study. Data sharing is not applicable to this article.

## Supplementary Materials

The following supporting information can be downloaded at: https://www.mdpi.com/article/10.3390/jcm15176697/s1. Includes a series of videos presenting how the selected clinical examination tests are performed in both healthy and RCT-affected shoulders. Tests include the belly press test, which is positive in the right shoulder (1.); bear hug test, with a positive result in the affected right shoulder (2.1.) and negative in the healthy shoulder (2.2.); lift-off test, with a positive result in the right shoulder (3.1.), negative in the healthy left shoulder (3.2.) and a modification of the lift-off test (3.3.), with a video presenting a comparison between the RCT-affected right shoulder and the healthy left shoulder, and the patient reporting lack of strength in the diseased joint; and empty can test, which is positive in the right shoulder (4.1.) and negative in the healthy left shoulder (4.2.). For detailed test descriptions, please see Section 2.1.

## Abbreviations

The following abbreviations are used in this manuscript:

RC	Rotator Cuff
RCT	Rotator Cuff Tear
ROM	Range of Motion
SPADI	Shoulder Pain and Disability Index
WOSI	Western Ontario Shoulder Instability Index
CMS	Constant–Murley Score
VAS	Visual Analogue Scale
MRI	Magnetic Resonance Imaging
US	Ultrasound
MRA	Magnetic Resonance Arthrography
PRP	Platelet-Rich Plasma
MT	Manual Therapy
DM	Diabetes Mellitus (type 2)
AAOS	American Academy of Orthopaedic Surgeons
SECEC	European Society for Surgery of the Shoulder and Elbow
ASSET	American Society of Shoulder and Elbow Therapists
LIPUS	Low-Intensity Pulsed Ultrasound
ESW	Extracorporeal Shock Waves
